# 

**Published:** 1830-01-01

**Authors:** 


### CONTENTS

OF THE

### MEDICO-CHIRURGICAL REVIEW.

### No. XXIII. JANUARY 1, 1830.

### REVIEWS.

I.

MEDICO-CHIRURGICAL TRANSACTIONS.

A Pathological Inquiry into the Secondary Effects of Inflammation of the Veins. By J. M. Arnott, Esq. .. .. .. .. .. .. .. .. .. .. .. .. 1

II.

An Essay upon the Treatment of the Deep and Excavated Ulcer, with Cases. By R. Anthony Stafford, Member of the Royal College of Surgeons, &c. .. 37

III.

Proposal of a Plan for the Investigation of the due Administration of Blood-letting. By Marshall Hall, M.D. .. .. .. .. .. .. .. .. .. .. .. .. 40

IV.

On the Mode of distinguishing Pregnancy from the Diseases which resemble it. By Robert Gooch, M.D. .. .. .. .. .. .. .. .. .. .. .. .. 42

V.

Elements of General Anatomy, containing an Outline of the Organization of the Human Body. By R. D. Grainger, Esq. .. .. .. .. .. .. .. .. 49

VI.

An Account of the Mode of Performing the Lateral Operation of Lithotomy. By Edward Stanley, Esq. .. .. .. .. .. .. .. .. .. .. .. .. .. 69

VII.

On some Symptoms in Children erroneously attributed to Congestion of the Brain. By Robert Gooch, M. D. .. .. .. .. .. .. .. .. .. .. .. .. 75

VIII.

MODERN WORKS ON MORBID ANATOMY.

1. Anatomie Pathologique du Corps Humain; ou Description avec Figures Lithographiées des diverses Altérations Morbides dont le Corps Humain est susceptible. Par J. Cruveilhier, Professeur d'Anatomie, &c. .. .. .. .. .. .. 79

II. Elements of General and Pathological Anatomy, adapted to the Present State of Knowledge in that Science. By David Cragie, M. D. .. .. .. .. .. *ib.*

IX.

On the Influence of Climate in the Prevention and Cure of Chronic Diseases, more especially of the Chest and Digestive Organs, &c. By James Clark, M.D. .. 90

X.

Lectures on Anatomy: interspersed with Practical Remarks. Vol. I. By B. B. Cooper, F.R.S. &c. .. .. .. .. .. .. .. .. .. .. .. .. 95

XI.

Eclectic Article on Pulmonary Tubercles or Tubercular Phthisis .. .. .. .. 97

XII.

A Treatise on Neuralgic Diseases, dependent on Irritation of the Spinal Marrow. By Mr. T. P. Teale .. .. .. .. .. .. .. .. .. .. .. .. .. .. 112

XIII.

Hospital Facts and Observations, &c. By Dr. J. L. Bardsley .. .. .. .. 129

XIV.

On Functional Disorders of the large Bowels and on Diseases which they induce. By James Annesley, Esq.. .. .. .. .. .. .. .. .. .. .. .. .. 138

### PERISCOPE.

1. Modern Medical Ethics; or State Maxims in Medicine. By Philo-Ethicus, *Artium Magister*, &c. .. .. .. .. .. .. .. .. .. .. .. .. .. 145
2. Case of supposed fatal Carditis. By Mr. Corbin .. .. .. .. .. .. .. .. 149
3. New Mode of treating Intestino-vaginal Fistula. By M. Casamayor .. .. 151
4. Prolapsus Ani treated after the Manner of Mr. Hey. By Dr. Macfarlane .. 154
5. Dr. Williams on the Sounds produced by the Action of the Heart; with Criticisms .. .. .. .. .. .. .. .. .. .. .. .. .. .. .. 155
6. Dysphagia from Diastasis of the Cornua of the Os Hyoides .. .. .. .. 159
7. Memoir on Iliac Abscesses and Tumours. By M. Teallier, M.D. .. .. .. 160
8. Case of Procidentia Uteri; with Remarks. By Mr. Knox.. .. .. .. .. .. 162
9. Fungus Hæmatodes found in the Fœtus.. .. .. .. .. .. .. .. .. .. 163
10. Case of Scirrhus Pylori and Ulceration of the Stomach. By Mr. Waldron, of Bath.. .. .. .. .. .. .. .. .. .. .. .. .. .. .. .. .. 164
11. Interesting Case of Supposed Carotid Aneurism, where the Artery was tied beyond the Tumour. By A. Montgomery, R.N... .. .. .. .. .. .. 165
12. Curious Case of Ascending Paralysis .. .. .. .. .. .. .. .. .. 168
13. Regulations of the Apothecaries' Company; with Remarks on the Apprenticeship System .. .. .. .. .. .. .. .. .. .. .. .. .. .. .. 169
14. The Rival Note Takers .. .. .. .. .. .. .. .. .. .. .. .. .. 193
15. Dr. Gooch on a Peculiar Hæmorrhage from the Uterus .. .. .. .. .. 195
16. Acute Purulent Ophthalmia following the Suppression of an habitual Leucorrhœa .. .. .. .. .. .. .. .. .. .. .. .. .. .. .. .. .. 196
17. Dr. Abercrombie on a Peculiar Delirium .. .. .. .. .. .. .. .. 197
18. Flour the Grand Panacea for Burns and Scalds .. .. .. .. .. .. .. 199
19. On Emphysema of the Lungs as a Cause of Asthma. By B. H. Coates, M.D. 200
20. Paraplegia after Apoplexy cured by the Moxa and Nux Vomica .. .. .. 203
21. Interesting Cases of Apoplexy, with Dissections. By Dr. Mills .. .. .. 204
22. Removal of Portions of Three Dorsal Vertebræ for Paralysis from Fracture .. 207
23. Lithotrity and Dilatation of the Urethra for Stone in the Female Bladder .. 209
24. Dr. Moran on the Uses of the Oil of Turpentine .. .. .. .. .. .. .. .. 211
25. Vaccination communicated from the Mother to the Fœtus in Utero .. .. 214
26. Partial Ramollissement of the Brain, accompanied with remarkable Symptoms, 215
27. Cases of Cynanche Trachealis. By Dr. Jackson .. .. .. .. .. .. .. .. 218
28. Dr. Monro on Medullary Sarcoma of the Stomach and Intestines .. .. .. 223
29. Lithotrity and Lithotomy .. .. .. .. .. .. .. .. .. .. .. .. .. 227

30. Observations on Pulmonary Consumption. By Dr. Parrish; with Remarks .. 241
31. Nothing new under the Sun—(Pompeian Instruments) .. .. .. .. .. 247
32. Dr. Bardsley on Iodine in Bronchocele, Scrofula, and Ascites .. .. .. .. 248
33. Gastric Irritation producing Symptoms resembling Apoplexy and Epilepsy .. 251
34. On Inflammation and Suppuration of the Spleen.. .. .. .. .. .. .. 252
35. Dr. Abercrombie on a peculiar Affection of the Pericranium, relieved by Division of the Membrane .. .. .. .. .. .. .. .. .. .. .. .. 256
36. Post-mortem Examination of the Kings of France, from Charles IX. to Louis XVIII .. .. .. .. .. .. .. .. .. .. .. .. .. .. .. .. .. 259
37. Distortion of the Face cured by Disease of the Brain .. .. .. .. .. .. 264
38. Dr. Monro on the Milt-like Tumour of Mucous Membranes .. .. .. .. 265

### CLINICAL REVIEW.

### Westminster Hospital.

1. Fracture of the Sternum, Clavicle, eight Ribs, &c. Diffuse Inflammation of the Scalp—Incisions.. .. .. .. .. .. .. .. .. .. .. .. 172
2. Lacerated Wound in the Bend of the Arm .. .. .. .. .. .. .. .. .. 174
3. Fatal Injury of the Knee-joint.. .. .. .. .. .. .. .. .. .. .. .. 230
4. Sympathetic Bubo, with a peculiar Eruption.. .. .. .. .. .. .. .. 231
5. Diseased Testicle .. .. .. .. .. .. .. .. .. .. .. .. .. .. 231
6. Medullary Sarcoma of the Ham—Affection of the Lungs and Liver.. .. .. 274
7. Ulceration of the Urethra—Extravasation of Urine, &c. .. .. .. .. .. 275
8. Compound Fracture of the Skull—Wound of the Brain, &c. .. .. .. .. 276
9. Psoriasis Diffusa.. .. .. .. .. .. .. .. .. .. .. .. .. .. .. 276
10. Diseased Penis—Amputation .. .. .. .. .. .. .. .. .. .. .. 277

### Winchester County Hospital.

1. Viper Bite—Severe Symptoms—Employment of Cupping-glasses, &c. .. .. 175
2. Cataracts alternating with Diabetes .. .. .. .. .. .. .. .. .. .. 176
3. Dislocation of the Clavicle forwards—Reduction at the end of 12 weeks.. .. 178
4. Dislocation of the Head of the Radius backwards .. .. .. .. .. .. 178
5. Renal Calculi—Laryngitis—Abscess external to the Larynx .. .. .. .. 179
6. Fungus Hæmatodes .. .. .. .. .. .. .. .. .. .. .. .. .. .. 235

### Hôpital Beaujon.

Discovery of a new Species of Inguinal Hernia .. .. .. .. .. .. .. .. 180

### Bath United Hospital.

Case of Extraordinary Constipation of the Bowels relieved by Medicine.. .. .. 181

### Naval Hospital of St. Petersburgh.

Removal of a Tumour from the Muscular Spiral Nerve .. .. .. .. .. .. 184

### St. George's Hospital.

Hysteria .. .. .. .. .. .. .. .. .. .. .. .. .. .. .. .. .. 186
A. Cough—Pain in the Side—Menorrhagia.. .. .. .. .. .. .. .. 186
B. Chlorosis—Pain in the Head and Chest .. .. .. .. .. .. .. .. 187
C. Pains in the Head, Chest, &c. .. .. .. .. .. .. .. .. .. .. 187
D. Pains in the Chest—Severe Cough, &c. .. .. .. .. .. .. .. .. 188

EDINBURGH SURGICAL HOSPITAL.

Quarterly Report. By J. Syme, Esq. .. .. .. .. .. .. .. .. .. .. .. 189
1. Excision of the Elbow-joint .. .. .. .. .. .. .. .. .. .. .. .. 189
2. Employment of the Actual Cautery .. .. .. .. .. .. .. .. .. .. 190
3. Excision of the Mamma .. .. .. .. .. .. .. .. .. .. .. .. 191
4. Hydrocele .. .. .. .. .. .. .. .. .. .. .. .. .. .. .. 192

ROYAL WESTMINSTER OPHTHALMIC HOSPITAL.

Mr. Guthrie on Oleum Terebinthinæ in internal Inflammation of the Eye .. .. 232

WORCESTER INFIRMARY.

1. Empyema with External Tumour .. .. .. .. .. .. .. .. .. .. .. 238
2. Employment of Strychnine in Hemiplegia .. .. .. .. .. .. .. .. 239
3. Supposed Hepatitis .. .. .. .. .. .. .. .. .. .. .. .. .. .. 240

HÔTEL DIEU.

1. The Rhinoplastic Operation for Destruction of the Lower Lip .. .. .. .. 268
2. Encysted Hydrocele of the Spermatic Cord .. .. .. .. .. .. .. .. 269
3. Vesico-vaginal Fistula, with the Observations of M. Dupuytren and Mr. Earle on that Disease .. .. .. .. .. .. .. .. .. .. .. .. .. .. 271

### MISCELLANIES.

1. Literary Property—Dr. Gooch and Dr. Burrows .. .. .. .. .. .. .. .. 278
2. Letter from Dr. Johnson to David Uwins, M.D. &c. .. .. .. .. .. .. 278
3. The late Dr. John Armstrong .. .. .. .. .. .. .. .. .. .. .. 281
4. Bibliographical Record .. .. .. .. .. .. .. .. .. .. .. .. .. 287

